# CYP4F22-Related Autosomal Recessive Congenital Ichthyosis Associated With Hirschsprung Disease and Bartter-Like Renal Manifestations

**DOI:** 10.14740/jmc5388

**Published:** 2026-09-04

**Authors:** Jamal Mohammed Alqahtani

**Affiliations:** Department of Dermatology, College of Medicine, Imam Abdulrahman Bin Faisal University, Dammam 31441, Saudi Arabia. Email: jamqahtani@iau.edu.sa

**Keywords:** Autosomal recessive congenital ichthyosis, *CYP4F22*, Congenital ichthyosiform erythroderma, Hirschsprung disease, Bartter syndrome, Collodion baby

## Abstract

Autosomal recessive congenital ichthyosis (ARCI) is a heterogeneous group of inherited cornification disorders caused by defects in epidermal barrier formation. Mutations in *CYP4F22* are an uncommon cause of ARCI and are associated with variable phenotypes including lamellar ichthyosis (LI) and congenital ichthyosiform erythroderma (CIE). This is a report of a genetically confirmed case of *CYP4F22*-related ARCI in a child born to consanguineous parents who presented with collodion membrane at birth followed by persistent ichthyosiform scaling and palmoplantar keratoderma. Genetic analysis identified a homozygous pathogenic *CYP4F22* variant, c.1303C>T p.(His435Tyr). In addition to cutaneous findings, the patient had Hirschsprung disease managed surgically during infancy and was followed by pediatric nephrology for Bartter-like manifestations associated with hypokalemia and bilateral renal stones. This case highlights the importance of recognizing phenotypic heterogeneity of *CYP4F22*-associated ARCI and describes unusual extracutaneous manifestations in association with this rare genodermatosis.

## Introduction

Autosomal recessive congenital ichthyosis (ARCI) represents a distinct group of genetic disorders affecting the keratinization process which is characterized by generalized scaling, variable erythema, and epidermal barrier dysfunction. Clinical manifestations varied from the most severe and often life-threatening harlequin ichthyosis phenotype to milder phenotypes such as congenital ichthyosiform erythroderma (CIE), lamellar ichthyosis (LI), and self-improving collodion ichthyosis [1].

Although ARCI is linked to multiple genes involved in epidermal barrier formation and keratinization, mutations in *CYP4F22* are recognized as a less frequent cause compared with other ARCI-associated genes. At the molecular level, *CYP4F22* encodes a cytochrome P450 enzyme involved in ω-hydroxylation of ultra-long-chain fatty acid, an essential step in acylceramide synthesis and corneocyte lipid envelope formation [2]. When this pathway is impaired, epidermal barrier formation becomes defective, resulting in abnormal keratinization and cornification. These molecular defects may manifest clinically as collodion membrane at birth, palmoplantar keratoderma, scaling, and variable erythema [3].

Recent studies have expanded the phenotypic spectrum associated with *CYP4F22* mutations, including reports describing ocular involvement and inflammatory pathway dysregulation [4, 5]. However, extracutaneous systemic manifestations remain rarely reported. Herein, we describe a genetically confirmed case of *CYP4F22*-related ARCI associated with Hirschsprung disease and Bartter-like renal manifestations.

## Case Report

A boy born to consanguineous parents presented at birth with generalized skin involvement described as a taut, shiny membrane covering the body, consistent with a collodion membrane phenotype. He was a second child with an unaffected older sibling and no reported similar skin disease among family members. Detailed documentation regarding associated neonatal complications was limited. Initial management was supportive, with attention to skin hydration and barrier protection.

During the early postnatal period, the collodion membrane gradually desquamated. However, this was followed by the development of persistent generalized skin dryness and ichthyosiform scaling. The persistence of generalized scaling from birth, in the context of parental consanguinity led to further dermatologic evaluation. Therefore, genetic testing was requested which showed a homozygous pathogenic variant in *CYP4F22*, c.1303C>T p.(His435Tyr), confirming the diagnosis of *CYP4F22*-related ARCI. This identified mutation has previously been reported in association with ARCI phenotypes [6]. Genetic testing for the identified *CYP4F22* variant was not performed in the patient’s parents or sibling.

**Figure 1:**
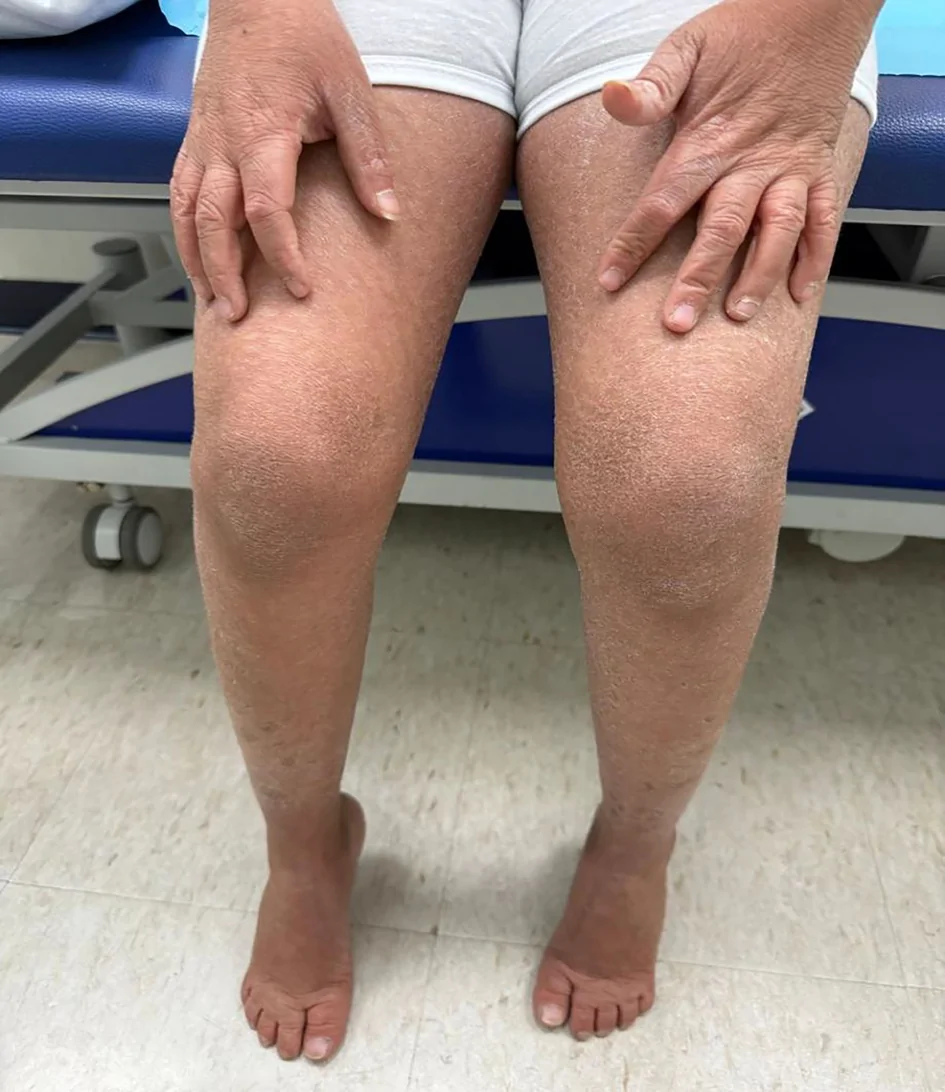
Diffuse fine scales involving both thighs and legs, without marked active erythema.

**Figure 2:**
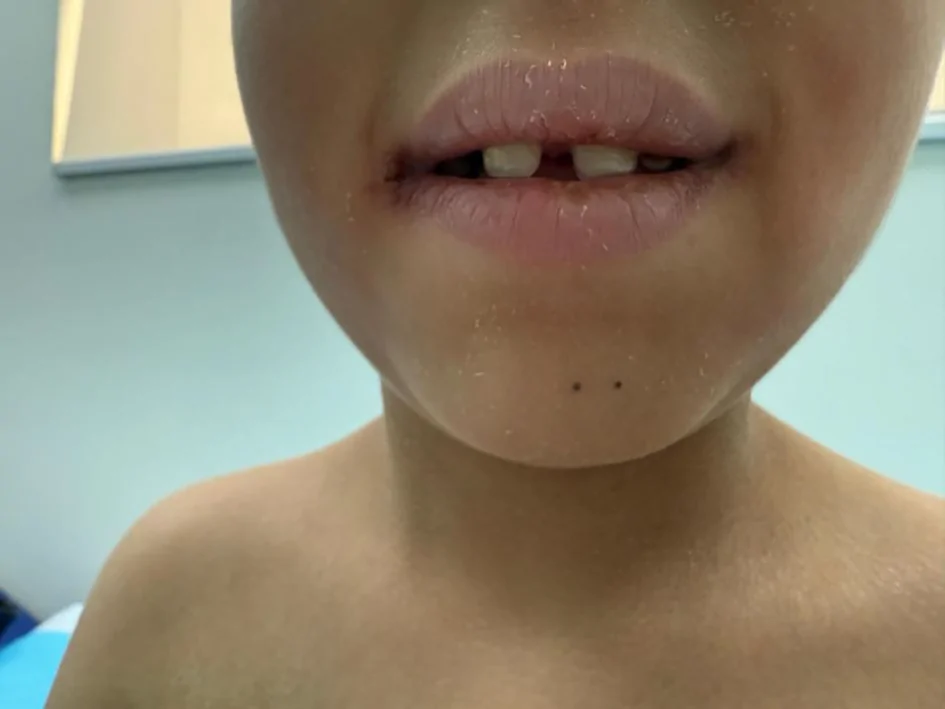
Perioral fine scaling and dryness.

**Figure 3:**
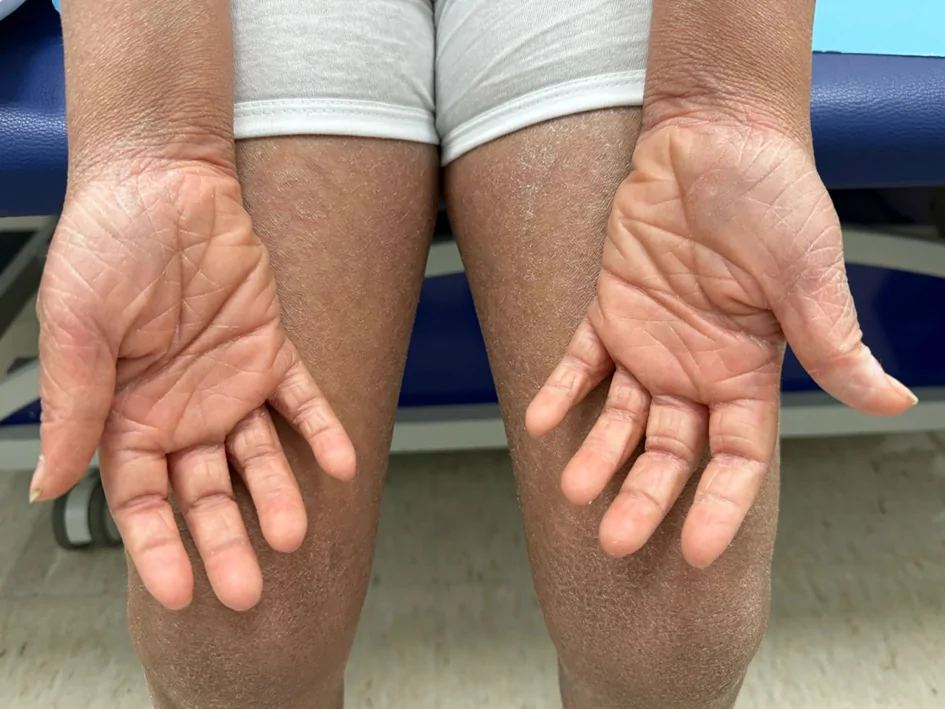
Mild palmar keratoderma with hyperlinearity. Diffuse fine scaling is also evident over the lower limbs.

**Figure 4:**
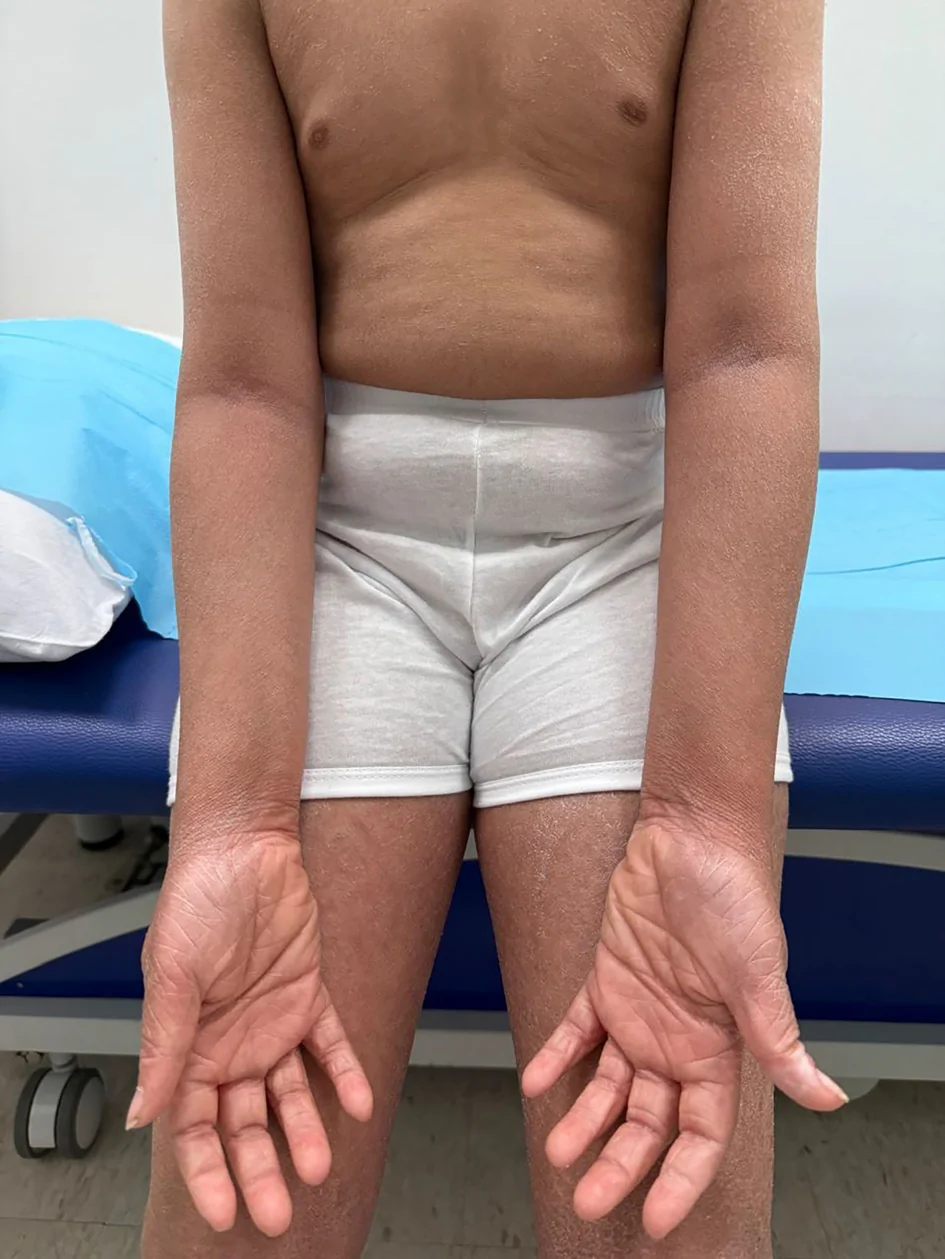
Diffuse fine scaling over the abdomen, arms, thighs, and legs, with relative absence of active erythema.

**Figure 5:**
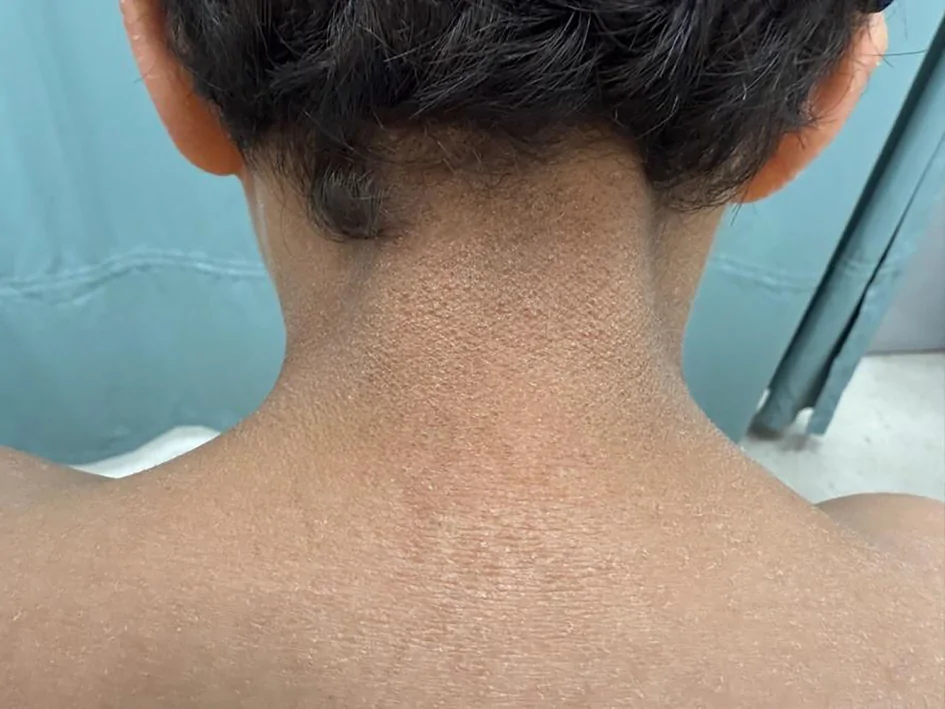
Ichthyosiform scaling, more apparent over the nape of the neck and upper trunk.

The patient was subsequently followed in the dermatology clinic as a case of CIE. On follow-up examination, the patient had diffuse fine whitish ichthyosiform scaling involving the trunk and extremities, with accentuation over the thighs, legs, forearms, and upper back. The palms showed hyperlinearity with mild palmoplantar keratoderma. Perioral dryness and fine facial scaling were also noted (Figs. 1–5). There was no significant active erythema, blistering, or erosions. The patient was managed conservatively with topical emollients and 20% urea cream, with partial symptomatic improvement.

In addition to the dermatologic manifestations, the patient had a history of Hirschsprung disease diagnosed later in childhood, around school age, following a prolonged history of chronic constipation and poor weight gain. The patient was managed by the pediatric surgery team and underwent surgical resection of the aganglionic segment of the colon, with subsequent improvement in gastrointestinal symptoms and weight gain after surgery. The patient was also followed by a pediatric nephrology team as a case of Bartter-like renal manifestations including recurrent hypokalemia requiring potassium supplementation and bilateral non-obstructing renal stones on serial ultrasonography. Because of the persistent hypokalemia and associated renal findings, the patient underwent nephrology-focused genetic testing. The analysis did not identify any clinically relevant sequence variants or copy number variations in the tested renal-associated genes. Accordingly, Bartter syndrome remained unconfirmed at the molecular level, and the renal and electrolyte abnormalities were regarded as Bartter-like manifestations managed with ongoing nephrology care and supportive electrolyte therapy.

Currently, the patient is on regular follow-up with a dermatologic clinic, and the family was counseled regarding the chronic nature of the disease and the need for long-term skin barrier maintenance were discussed with the family. The treatment plan focused on a regular application of bland emollients, keratolytic therapy with urea-containing preparations, avoidance of excessive skin dryness, and monitoring for secondary complications such as fissuring, irritation, or infection. Although erythema improved over time, persistent scaling and palmoplantar keratoderma remained evident.

### Genetic testing result

CentoSkin testing, including sequencing and next generation sequencing (NGS)-based copy number variation analysis, revealed a homozygous pathogenic variant in the *CYP4F22* gene. A genetic diagnosis of ARCI type 5 was confirmed (mutated gene: *CYP4F22*; variant: NM_173483.3:c.1303C>T; amino acid change: p.(His435Tyr)). No other clinically relevant sequence variants or copy number variations were identified in the remaining analyzed panel genes.

The *CYP4F22* variant c.1303C>T p.(His435Tyr) is a missense variant that results in an amino acid substitution from histidine to tyrosine at position 435. This variant has previously been reported as disease-causing in association with LI with hyperlinearity of the palms and soles. It is also listed in ClinVar as pathogenic. Based on CENTOGENE and ACMG recommendations, the variant was classified as pathogenic. Pathogenic variants in *CYP4F22* are associated with ARCI, a heterogeneous group of disorders of keratinization characterized primarily by generalized abnormal skin scaling.

## Discussion

This report highlights an unusual presentation of genetically confirmed *CYP4F22*-related ARCI in a boy born to consanguineous parents. The patient exhibited characteristic features of the disease, including a collodion membrane at birth, persistent ichthyosiform scaling, and palmoplantar keratoderma, together with the homozygous *CYP4F22* c.1303C>T p.(His435Tyr) variant. In addition to these typical cutaneous manifestations, the patient developed Hirschsprung disease and Bartter-like renal abnormalities, which represent an unusual clinical combination in *CYP4F22*-related ARCI.

ARCI comprises a clinically and genetically heterogeneous group of disorders characterized by abnormal cornification and impaired epidermal barrier function [1]. Mutations in *CYP4F22* represent an uncommon cause of ARCI and are estimated to account for approximately 8% of cases [3]. The encoded enzyme participates in ultra-long-chain fatty acid ω-hydroxylation, which is a critical step in corneocyte lipid envelope assembly and acylceramide synthesis [2]. Impairment in this pathway results in defective epidermal barrier function and development of ichthyosis.

More recently, a gene-based classification of non-syndromic epidermal differentiation disorders (nEDDs) has been proposed, in which *CYP4F22*-related disease is designated as *CYP4F22*-nEDD. This updated classification emphasizes the underlying genetic defect and molecular pathogenesis rather than traditional phenotype-based terminology [7].

The clinical spectrum with *CYP4F22* mutations showed a variable phenotypic presentation that includes CIE, LI, and self-improving collodion ichthyosis. Reported clinical manifestations frequently include collodion membrane at birth, scaling, palmoplantar keratoderma or hyperlinearity, and variable erythema [3, 8]. In our patient, multiple characteristic features were observed including neonatal collodion presentation, persistent truncal scaling, and palmoplantar keratoderma.

The identified homozygous c.1303C>T p.(His435Tyr) variant has previously been reported as pathogenic in association with ARCI [6]. Overlapping features between CIE and LI are well recognized in *CYP4F22*-associated disease, and histopathology typically demonstrates non-specific findings [3].

Additional notable extracutaneous features in our patient were the co-existence of Hirschsprung disease requiring surgical intervention and clinically diagnosed Bartter-like manifestations associated with hypokalemia and recurrent bilateral non-obstructing renal stones. To the best of our knowledge, no established association between *CYP4F22* mutations and either Hirschsprung disease or Bartter-like manifestations has been reported. *CYP4F22* deficiency primarily affects epidermal lipid metabolism and skin barrier formation, whereas the gastrointestinal and renal findings involve distinct physiological pathways. Therefore, a direct causal relationship cannot currently be established. In the setting of parental consanguinity, the co-existence of these conditions may reflect multiple inherited disorders rather than a unified syndromic entity. Nevertheless, the presence of unusual extracutaneous manifestations broadens the clinical context in which *CYP4F22*-related ichthyosis may be encountered. This also highlights the importance of multidisciplinary follow-up, particularly when gastrointestinal and renal findings co-exist with a genetically confirmed congenital ichthyosis.

Emerging evidence has highlighted inflammatory pathway activation involving IL-17 and IL-23 signaling in ARCI and has demonstrated therapeutic responses to biologic agents in selected patients [5]. Additionally, ultrastructural studies have shown that *CYP4F22* deficiency leads to impaired acylceramide synthesis and disruption of corneocyte lipid envelope formation, providing a mechanistic basis for the epidermal barrier dysfunction observed in these patients [2].

Consanguinity plays an important role in autosomal recessive genodermatoses, including ARCI, as it increases the probability of homozygosity for rare pathogenic variants. In a cohort study of 125 consanguineous families with ARCI, Youssefian et al detected biallelic mutations in most families, most of which were homozygous, emphasizing the impact of consanguinity on the genetic basis of ARCI [9]. In addition, although *CYP4F22*-related ARCI is generally considered a non-syndromic form of ichthyosis, the presence of extracutaneous manifestations warrants thorough clinical and genetic assessment. Aubry et al illustrated this diagnostic complexity in a consanguineous family in which ichthyosis and ocular abnormalities initially suggested a syndromic form of ichthyosis, but exome sequencing ultimately identified two separate autosomal recessive conditions [10]. Thus, the Hirschsprung disease and Bartter-like renal features observed in the present case may represent associated extracutaneous findings, a second recessive disorder, or co-incidental comorbidities, and broader genomic testing, including whole-exome sequencing, may help clarify the underlying basis for these additional manifestations.

In conclusion, we present a genetically confirmed case of *CYP4F22*-related ARCI associated with Hirschsprung disease and Bartter-like manifestations. This case further broadens the phenotypic spectrum associated with *CYP4F22* mutations and highlights the need of comprehensive clinical and genetic evaluation when extracutaneous manifestations are present.

### Learning points

*CYP4F22*-related ARCI can present at birth with a collodion membrane, followed by persistent generalized ichthyosiform scaling and palmoplantar keratoderma. Consanguinity represents an important risk factor for autosomal recessive genodermatoses and should raise suspicion for homozygous pathogenic variants, prompting early genetic evaluation and family counseling. Although *CYP4F22*-associated ichthyosis is usually considered non-syndromic, the presence of extracutaneous manifestations should raise the possibility of syndromic ichthyosis, an additional autosomal recessive disorder, or co-incidental comorbidity. Therefore, the co-existence of ichthyosis with Hirschsprung disease and Bartter-like renal manifestations highlights the importance of multidisciplinary assessment and careful genotype–phenotype correlation.

## Data Availability

All data generated or analyzed during this study are included in this published article. Additional information is available from the corresponding author upon reasonable request, in accordance with patient confidentiality and ethical considerations.
